# Straw type governs methane-cycling microbiomes and CH_4_ emissions in paddy soils via abiotic and biotic interactions

**DOI:** 10.3389/fmicb.2025.1750602

**Published:** 2026-01-16

**Authors:** Yanbo Wang, Yijia Zhang, Yang Ji, Yanfang Feng, Zhaozhong Feng

**Affiliations:** 1 Key Laboratory of Ecosystem Carbon Source and Sink, China Meteorological Administration (ECSS-CMA), School of Ecology and Applied Meteorology, Nanjing University of Information Science and Technology, Nanjing, China; 2 Key Laboratory of Agro-Environment in Downstream of Yangtze Plain, Ministry of Agriculture and Rural Affairs, Institute of Agricultural Resources and Environment, Jiangsu Academy of Agricultural Sciences, Nanjing, China

**Keywords:** methane cycling, methane oxidation, methanogenesis, paddy soil, straw incorporation

## Abstract

Straw incorporation is widely practiced in rice paddies to enhance soil fertility and crop yield, yet its effects on methane (CH_4_) emissions remain uncertain due to complex microbial and soil interactions. We conducted a soil column experiment with a no-straw control (CK) and amendments of rice (RS), wheat (WS), and maize (MS) straw. Seasonal CH_4_ fluxes, soil properties, CH_4_-cycling microbes, and abundances of *mcrA* and *pmoA* were analyzed across four rice growth stages. RS, WS, and MS significantly increased cumulative CH_4_ emissions by 15.7, 14.2, and 18.6 g m^–2^, respectively, with no significant differences among straw types. Soil pH significantly decreased under straw treatments, while rice grain yield significantly increased by 13.7–25.9%. Partial least squares path modeling (PLS-PM) analysis indicated that CH_4_ emissions were strongly negatively influenced by soil properties and microbial community composition. Among all the soil properties, the reduction in soil pH resulting from straw incorporation was the most significant factor increasing CH_4_ emissions. Microbial biomass carbon (MBC) contributed to CH_4_ emission variations, with its effect primarily driven by growth stage differences. Straw incorporation simultaneously stimulated a shift in the methanogenic toward *Methanosarcinaceae* and a shift in the methanotrophic toward *Methylocystaceae*. In contrast, the enhanced methane oxidation was insufficient to counterbalance the methanogenesis, causing increased net CH_4_ emissions. Although CH_4_ emissions were comparable among straw types, wheat straw achieved the largest (non-significant) yield increase, suggesting that wheat straw incorporation may offer a favorable balance between yield enhancement and CH_4_ emissions, warranting further field-based verification.

## 1 Introduction

CH_4_ is the second most influential greenhouse gas, with a 100-year global warming potential (GWP) 28 times that of CO_2_ (IPCC, 2022). Since the Industrial Revolution, atmospheric CH_4_ concentrations have risen continuously, reaching a record 1934 ± 2 ppb by 2023, equivalent to 265% of preindustrial levels (WMO, 2024), and contributing roughly 0.5 °C to the global mean surface temperature increase of approximately 1.09 °C (range: 1.0 °C–1.2 °C) since industrialization (IPCC, 2023). Agriculture remains the dominant contributor to anthropogenic CH_4_ emissions (Zhu and Li, 2025), responsible for an estimated 68% of global human-sourced CH_4_ emissions (Saunois et al., 2020). Within this sector, rice cultivation alone contributes 9–10% of atmospheric CH_4_ emissions (FAO, 2023) and nearly half (48%) of the total greenhouse gas emissions originating from paddy systems (Qian et al., 2023). These statistics underscore the necessity of incorporating CH_4_ mitigation into broader climate policy frameworks.

China, as a leading global agricultural producer, generates substantial quantities of crop residues annually from its major food and cash crops. By the end of 2020, the overall utilization rate of crop straw had risen to 87.6%, with direct field incorporation contributing 62.1% (Sun et al., 2022). Straw incorporation has become a pivotal sustainable practice to enhance soil fertility and reduce reliance on chemical fertilizers (Zhao et al., 2025). However, by introducing abundant labile carbon, such as cellulose and hemicellulose, this practice profoundly influences soil carbon cycling and microbial processes (Conrad, 2007; Jiang et al., 2025).

Straw incorporation serves as a major carbon source that fuels anaerobic decomposition in flooded paddy soils, thereby promoting CH_4_ production (Zhao et al., 2023). Isotope tracing studies have demonstrated that approximately 20% of the CH_4_ produced in rice paddies is derived from the carbon in incorporated straw (Yuan et al., 2012). The input of labile substrates stimulates microbial activity, increases *mcrA* gene abundance, and promotes CH_4_ production (Liu et al., 2018; Mei et al., 2025). Meanwhile, CH_4_ oxidation, mediated by methanotrophs, also responds to changes in substrate availability and soil redox conditions (Jiang et al., 2019). Thus, the balance between methanogenesis and methanotrophy ultimately determines net CH_4_ flux in paddy fields (Xu et al., 2020; Wang et al., 2025).

However, not all straw types affect CH_4_ emissions in the same way, as their chemical composition and decomposition dynamics differentially influence the abundance and community structure of methanogens and methanotrophs. Maize, rice, soybean, and wheat residues have different chemical compositions and decay rates, thereby modulating CH_4_ production and oxidation (Choudhary et al., 2024). For instance, high-lignin straw (e.g., maize stover) undergoes a prolonged phase of aerobic and facultative anaerobic decomposition, which delays the availability of substrates required for methanogenic communities (Liang et al., 2017), but when retained at high rates ( ≥ 5 Mg ha^−1^), it can ultimately increase CH_4_ emissions by approximately 25% (Battaglia et al., 2022). Conversely, the rapid decomposition traits, low C/N ratio, and release of methanogenesis-inhibiting metabolites (e.g., pterins and flavonoids) in rapeseed straw collectively result in a significantly smaller magnitude of CH_4_ emissions enhancement in paddy fields compared to wheat straw application (Wang et al., 2024). Critically, the influence of straw C/N ratio is often mediated by its association with labile carbon fractions. In soil systems, lower straw C/N ratios have been associated with increased labile carbon fractions and enhanced abundances of *mcrA* and *pmoA* genes, further modulating methanogenic communities (Chen et al., 2024). Moreover, mechanistic insights from anaerobic digestion (AD) studies suggest that lower C/N ratios can enrich hydrogenotrophic methanogens (e.g., *Methanobacterium*) and versatile *Methanosarcina*, promoting hydrogenotrophic pathways (Zheng et al., 2021), whereas other AD studies report that low C/N ratios favor acetoclastic methanogenesis, with higher C/N ratios shifting the pathway toward hydrogenotrophy (Zheng et al., 2022). Methanotrophs exhibit similar sensitivity, with low C/N environments tending to enrich Type I methanotrophs (e.g., *Methylomonas*) over Type II groups (e.g., *Methylocystis*) (Zhou et al., 2020b). These discrepancies are likely attributable to differences in substrate biochemical composition and reaction conditions rather than system type. However, paddy soils are more complex and dynamic, with periodic wet–dry cycles and multiple interacting factors. How different straw type regulate the structure of methanogenic and methanotrophic communities in such systems, and thereby influence CH_4_ emissions, remains largely unexplored.

Based on this background, we hypothesize that different straw types have distinct effects on CH_4_ emissions from paddy soils, mediated by straw-specific responses of CH_4_-cycling microbial communities. To test this hypothesis, we conducted soil column experiments using paddy soil from Yixing City, Jiangsu Province. Four treatments were established: CK - control, RS - rice straw, WS - wheat straw, and MS - maize straw. We measured CH_4_ fluxes, evaluated key soil properties, and characterized the composition and diversity of methanogenic and methanotrophic communities at major rice growth stages. This study elucidates how various straw types regulate CH_4_ emissions by altering soil carbon inputs and microbial functions, providing a microbial perspective to support improved CH_4_ accounting and straw management in rice agriculture.

## 2 Materials and methods

### 2.1 Experimental design

We conducted a soil column experiment in the modern greenhouse at the Jiangsu Academy of Agricultural Sciences (JAAS), Nanjing, China. The rice cultivar “Nanjing 46” was transplanted in June 2020 and harvested in November 2020. Soil was collected from a single-season paddy field in Yixing, Jiangsu Province, China. The soil was a *Gleyi-Stagnic Anthrosol* (CRGCST, 2001). The soil was carefully sampled from three distinct layers (0–20 cm, 20–40 cm, and 40–60 cm). Each layer was thoroughly mixed separately to ensure uniformity within each layer. A total of 35 kg of homogenized soil from these layers was then packed into each soil column (inner diameter 0.30 m, height 0.50 m), with each layer of soil packed in succession, preserving the natural stratification to simulate paddy conditions. The initial physicochemical properties of the soil were as follows: soil pH was 6.38, determined using the potentiometric method (Zheng et al., 2024); organic matter content was 29.2 g kg^–1^, determined by the potassium dichromate oxidation method (Walkley and Black, 1934); cation exchange capacity was 22.61 °c mol kg^–1^, measured by the ammonium acetate method (Schollenberger and Simon, 1945); total nitrogen content was 1.72 g kg^–1^, measured using an elemental analyzer (Jimenez and Ladha, 1993); available phosphorus content was 23.09 mg kg^–1^, measured by the Olsen method (Battisti et al., 2022); and available potassium content was 159.28 mg kg^–1^, determined by the ammonium acetate method (Angon et al., 2023). Four treatments were established: a no-straw control (CK), rice straw (RS), wheat straw (WS), and maize straw (MS). Straw was applied at 0.8% of dry soil (equivalent to 8 t ha^–1^), with three replicates per treatment, for a total of 12 soil columns. All straw materials were air-dried, cut into 1–2 cm segments, placed in 15 × 20 cm, nylon bags (350-mesh), and buried at 5–10 cm depth in the center of each soil column. The chemical properties of the straw materials are summarized in Supplementary Table 1. Fertilization consisted of 240 kg N ha^–1^ split 2:1:1 ratio for basal, tillering, and panicle applications, and 96 kg P_2_O_5_ ha^–1^ and 192 kg K_2_O ha^–1^ applied once as basal phosphorus and potassium. Water management followed a local intermittent irrigation regime: continuous flooding (30 June–29 July), midseason drainage (30 July–12 August), alternating wet-dry cycles (12 August–14 October), and final drainage until harvest (15 October–9 November).

### 2.2 Gas and soil sample collection and determination

CH_4_ fluxes and their seasonal variations during the rice growing season, under various straw incorporation treatments, were quantified using the closed static dark chamber method coupled with gas chromatography (Li et al., 2025). Before sampling, opaque PVC chambers (25 cm × 21 cm × 80 cm) were placed on each soil column and sealed with a thin water film to ensure airtightness. A small fan mixed the headspace air, and chamber temperature was recorded at each sampling event. Sampling was conducted between 08:00 and 10:00. Four headspace gas samples (20 mL) were collected at 0, 15, 30, and 45 min after chamber closure. CH_4_ concentrations were determined using a Shimadzu GC-12A gas chromatograph equipped with a hydrogen flame ionization detector (FID). Gas sampling was performed every other day during the tillering, jointing, heading, and maturing stages, and every 2 weeks during the final 2 months before harvest.

The CH_4_ flux was calculated as follows:


$$F=\rho \times \frac{V}{A}\times \frac{d\,c}{d\,t}\times \frac{273}{273+T}$$

where *F* is the CH_4_ flux (mg m^–2^ h^–1^), ρ is the density of CH_4_ at standard conditions (0.714 kg m^–3^), *V* is the effective headspace volume of the chamber (m^–3^), *A* is the soil surface area covered by the chamber (m^–2^), *dc*/*dt* is the rate of change in gas concentration over time (μmol mol^1^ h^–1^), and *T* is the temperature inside the chamber at sampling (°C).

The seasonal cumulative CH_4_ emissions (T) was calculated as follows:


$$T=\Sigma \,\frac{\left(F_{i+1}+F_{i}\right)\times \left(D_{i+1}-D_{i}\right)\times 24}{2\times 1000}$$

where *T* is the cumulative seasonal CH_4_ emissions (g m^–2^), *F*_*i*_ and *F*_*i*+1_ denote the mean CH_4_ fluxes (mg m^–2^ h^–1^) at the i and i+1 sampling events, and *D*_*i*_ and *D*_*i*+1_ denote the sampling dates at the i and i+1 events (d).

Surface soil (0–10 cm depth) was collected at four key rice growth stages, tillering, jointing, heading, and maturing stages, along with surface water samples from the paddy. Following filtration, NH_4_^+^-N and NO_3_^–^-N concentrations in the water were determined using a SKALAR SAN++ SYSTEM flow analyzer (Skalar, the Netherlands). Soil pH was measured with a Mettler Toledo Five Easy Plus pH meter after shaking soil in CO_2_-free deionized water (1:2.5) and centrifuging for 5 min; the supernatant was used for analysis. Microbial biomass carbon (MBC) and nitrogen (MBN) were measured using chloroform fumigation extraction combined with total organic carbon analysis.

### 2.3 DNA extraction, PCR amplification, sequencing and data processing

At four key growth stages of rice (tillering, jointing, heading, and maturing), 0.5 g of surface soil samples were collected for DNA extraction. Genomic DNA was extracted using the Fast DNA SPIN Kit (MP Biomedicals, Eschwege, Germany). The quality and concentration of DNA were determined by 1.0% agarose gel electrophoresis and a NanoDrop2000 spectrophotometer (Thermo Scientific, United States) and kept at −80 °C prior to further use.

The *pmoA* and *mcrA* genes were targeted to profile methanotrophic and methanogenic communities, respectively. PCR amplification was performed using barcoded primers A189f/Mb661R for *pmoA* (Thao et al., 2024) and MLfF/MLrR for *mcrA* (Ren et al., 2020). Detailed PCR conditions, purification procedures, and library preparation are provided in the Supplementary Methods. Amplicon libraries were sequenced on an Illumina NextSeq 2000 platform (Shanghai Meiji Biomedical Technology Co., Ltd.), using paired-end reads. High-quality merged sequences were clustered into operational taxonomic units (OTUs) at 97% sequence similarity using UPARSE v7.1^1^, with chimera sequences removed during clustering. The average Good’s coverage per sample remained at 99.98%, indicating sufficient sequencing depth to characterize microbial communities. Taxonomic classification of *pmoA* and *mcrA* gene OTUs was conducted using the RDP Classifier (version 2.11)^2^^,^^3^; against the fgr/*pmoA*_202012 and fgr/*mcrA*_202012 reference databases, with a confidence threshold of 70%. Community composition was summarized at multiple taxonomic levels for each sample. The raw data have been deposited in the NCBI Sequence Read Archive under the accession numbers PRJNA1254631 (*mcrA* gene) and PRJNA1254505 (*pmoA* gene).

### 2.4 Statistical analyses

Data processing and visualization were conducted using the software listed below. Microsoft Excel 2021 was used for data organization and descriptive statistics. Prior to formal statistical testing, the assumptions of parametric tests were verified for each dataset. Homogeneity of variances was assessed using Levene’s test, and residual normality was evaluated using the Shapiro–Wilk test. Based on these diagnostics, the following tests were applied: (i) one-way ANOVA with Tukey’s HSD for data meeting assumptions; (ii) Welch’s ANOVA with Games–Howell for heterogeneous variances; and (iii) Kruskal–Wallis with Dunn’s test for non-normal data. The normality and homogeneity of variance of each key response variable are presented in the Supplementary materials (Supplementary Table 2). Analysis of variance (ANOVA) on alpha diversity indices was performed using JMP 10, followed by Tukey’s HSD test for multiple comparisons at a significance level of *P* < 0.05. Pearson’s correlation coefficients were calculated with two-tailed tests at significance levels of 0.05, 0.01, and 0.001. Figures were prepared in Origin 2021. Partial least squares path modeling (PLS-PM) was implemented in RStudio 4.3.2 to evaluate relationships among physicochemical properties, microbial metrics, and CH_4_ emissions. Variance Inflation Factor (VIF) were calculated to assess multicollinearity among manifest variables before PLS-PM. Variables with high VIF values and low outer loadings (e.g., MBN) were removed to ensure model validity. Additional microbiome analyses were conducted on the Meiji BioCloud platform^–3^, and differential taxa were tested using the Kruskal–Wallis test. Principal coordinate analysis (PCoA) based on Bray-Curtis dissimilarity was used to evaluate similarities in microbial communities among samples. When the Kruskal–Wallis test indicated significance, *post hoc* pairwise comparisons among treatments were performed using Dunn’s test. Linear discriminant analysis effect size (LEfSe) identified taxa with differential abundance between groups from phylum to genus levels, using a “one-against-all” strategy, with an LDA score threshold >2.0 and *P* < 0.05. Redundancy analysis (RDA) was used to evaluate associations between soil variables and methanogenic and methanotrophic community composition.

## 3 Results

### 3.1 CH_4_ emissions, soil properties and microbial abundance

**FIGURE 1:**
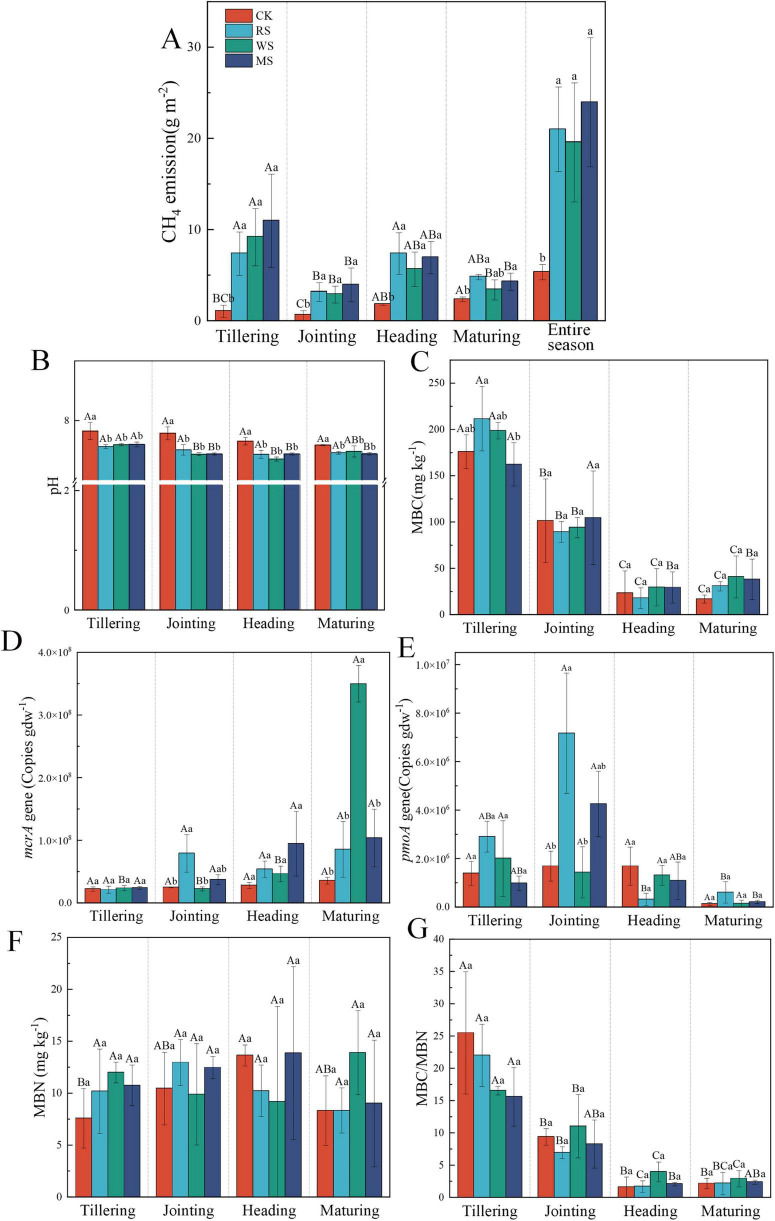
CH_4_ emissions, soil properties and microbial abundance. Changes in cumulative CH_4_ emissions **(A)**, soil pH **(B)**, MBC **(C)**, *mcrA* gene copies **(D)**, *pmoA* gene copies **(E)**, MBN **(F)**, and MBC/MBN ratio **(G)** across treatments during rice growth stages. Different lowercase letters indicate significant differences among treatments within the same growth stage (*P* < 0.05), whereas different uppercase letters indicate significant differences among growth stages within the same treatment (*P* < 0.05). RS, WS, and MS denote rice, wheat, and maize straw incorporation, respectively.

Across all treatments, CH_4_ emissions peaked during early growth stages, particularly after basal and tillering fertilizer applications, and declined sharply after field drainage (Supplementary Figure 1). Maximum fluxes occurred just before drainage, with MS showing the highest peak (28.4 mg m^–2^ h^–1^), followed by WS (21.6 mg m^–2^ h^–1^) and RS (20.2 mg m^–2^ h^–1^). Relative to CK, total CH_4_ emissions increased by 15.7 g m^–2^, 14.2 g m^–2^, and 18.6 g m^–2^ under RS, WS, and MS, respectively (*P* < 0.05), whereas differences among the three straw treatments were not significant (*P* > 0.05) (Figure 1A). Across all growth stages, CH_4_ emissions were consistently higher during the tillering and heading stages compared to the jointing and maturity stages. Emissions under RS, WS, and MS were significantly greater than those under CK at each stage (*P* < 0.05), consistent with the seasonal total patterns.

Straw incorporation significantly reduced soil pH (*P* < 0.05) (Figure 1B). MBC and MBN tended to increase during the early growth stages, although differences among treatments were not statistically significant (*P* > 0.05) (Figures 1C, F). Both MBC and the MBC/MBN ratio showed an overall decreasing trend as rice growing, with significantly lower values at heading and maturity than at tillering and jointing (*P* < 0.05), while heading and maturity did not differ significantly (*P* > 0.05) (Figure 1G). NH_4_^+^-N and NO_3_^–^-N contents generally decreased as the rice plants progressed through growth stages. Compared with CK, the WS and MS treatments significantly increased NH_4_^+^-N content at the tillering stage, whereas RS and MS significantly reduced NH_4_^+^-N content at the heading stage. The NO_3_^–^-N concentration was significantly lower under the RS, WS, and MS treatments than under CK at the tillering stage, whereas the MS treatment resulted in a significantly higher NO_3_^–^-N concentration than CK at the heading stage (*P* < 0.05) (Supplementary Figure 2). To facilitate an overall comparison among treatments, mean values across all growth stages were calculated for each treatment and provided in the Supplementary materials (Supplementary Figure 3). These averaged results showed that straw incorporation significantly changed soil pH compared with CK (*P* < 0.05), whereas MBC, MBN, MBC/MBN, NH_4_^+^-N and NO_3_^–^-N showed no significant treatment effects (*P* > 0.05). Copy numbers of the *mcrA* gene increased throughout the season, peaking at maturity (Figure 1D). WS induced the highest *mcrA* abundance, significantly exceeding other treatments (*P* < 0.05). In contrast, *pmoA* copy numbers varied less among treatments and growth stages. A significant difference among treatments was observed only at the jointing stage, where RS showed higher *pmoA* abundance than CK and WS (*P* < 0.05), whereas no significant treatment effects were detected at the other stages (*P* > 0.05) (Figure 1E). In addition, straw incorporation significantly increased rice grain yield compared with CK, with increases ranging from 13.7% to 25.9% (*P* < 0.05). Among the treatments, WS showed the highest increase (25.9%), followed by RS (22.9%) and MS (13.7%) (Supplementary Figure 4).

### 3.2 Shifts in the community diversity of methanogens and methanotrophs

The incorporation of different straw types significantly altered the α- and β-diversity of both methanogenic and methanotrophic communities, with effects varying by growth stage. For methanogens, Shannon and Simpson indices varied significantly among straw types (*P* < 0.05), whereas species richness (Chao1) remained largely unchanged (Supplementary Table 3). These diversity responses were strongly stage-dependent: at the tillering stage, MS increased Shannon diversity relative to CK and WS (*P* < 0.05), whereas by the jointing stage, RS exhibited the lowest Shannon index and the highest Simpson index (*P* < 0.05), indicating increasing dominance of a few taxa. In WS, the maturing stage was characterized by a marked decline in Shannon diversity and evenness (*P* < 0.05), accompanied by a higher Simpson index. Methanotrophic α-diversity showed similar stage-specific patterns (Supplementary Table 4). The strongest treatment effects occurred at the heading stage, where both RS and MS significantly reduced the Shannon index and increased the Simpson index compared to CK (*P* < 0.05). For RS, the Simpson index at the heading stage was also significantly higher than at the tillering stage.

**FIGURE 2:**
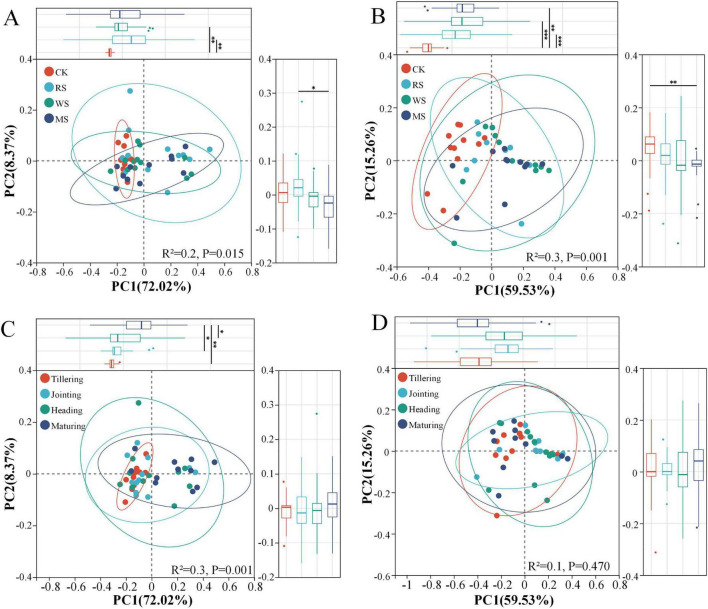
Principal coordinate analysis (PCoA) of methanogenic and methanotrophic community. Methanogenic communities under different treatments **(A)**, methanotrophic communities under different treatments **(B)**, methanogenic communities across distinct growth stages **(C)**, methanotrophic communities across distinct growth stages **(D)**. *, **, *** represent significant differences between treatments with *P* < 0.05, 0.01, and 0.001, respectively.

PCoA using Bray-Curtis differences was employed to assess how straw incorporation treatments and growth stages affected the methanogenic and methanotrophic communities at the family level. For methanogens, treatment groups (Figure 2A) and growth stages (Figure 2C) diverged significantly (*P* < 0.05) along the first two principal coordinate axes, which together explained 80.4 % of the total variance (PC1: 72.1%; PC2: 8.4%). Along PC1, the CK separated clearly from RS and WS (*P* < 0.05); the tillering stage separated clearly from jointing, heading and maturing stage, indicating substantial temporal changes in methanogenic communities. PC2 further distinguished RS from MS (*P* < 0.05), suggesting secondary treatment effects. Similarly, methanotrophic communities were significantly structured by treatment (*P* < 0.05), with PC1 accounting for 59.5% and PC2 for 15.3% (cumulative 74.8%). CK again segregated from all straw incorporation treatments along PC1 (*P* < 0.05) (Figure 2B), while PC2 specifically separated CK from MS (*P* < 0.05). In contrast, no significant differences were observed across growth stages (*P* = 0.47) (Figure 2D).

### 3.3 The OTUs of methanogens and methanotrophs across treatments and growth

Venn diagram analysis at the OTU level revealed distinct responses of methanogenic archaea and methanotrophic bacteria to straw incorporation. For the methanogens (Supplementary Figure 5A), a total of 301 core OTUs (46 %) were shared across all four treatments. Straw amendments introduced an additional 220 OTUs (34%) relative to the CK, of which 40 (6%), 30 (5%), and 47 (7%) were uniquely associated with RS, WS, and MS, respectively. In contrast, the methanotrophs exhibited a markedly stronger response (Supplementary Figure 5B). Only 274 core OTUs (5%) were shared across treatments, while straw addition introduced 4499 new OTUs (75%). Among these, 1876 (31%), 245 (4%), and 1743 (29%) were uniquely detected in RS, WS, and MS, respectively.

The methanogenic community underwent dynamic compositional changes throughout the rice growing season. For the methanogens, a core set of 162 OTUs (34%) was shared across all four growth stages (Supplementary Figure 5C). Meanwhile, each stage also harbored unique OTUs: 10 (2%) at the tillering stage, 41 (9%) at jointing, 58 (12%) at heading, and 38 (8%) at maturing. In contrast, the methanotrophs showed much bigger differences over time (Supplementary Figure 5D). Only 307 OTUs (5%) were shared across the four growth stages. The maturing stage had the largest number of unique OTUs (2,900, 49%), followed by the tillering stage (544, 9%), jointing stage (731, 12%), and heading stage (261, 4%). Overall, both the different treatments and growth stages had a significant impact on the structure of CH_4_-cycling microbial communities, with methanotrophs being more strongly affected.

### 3.4 Community composition of methanogens and methanotrophs

**FIGURE 3:**
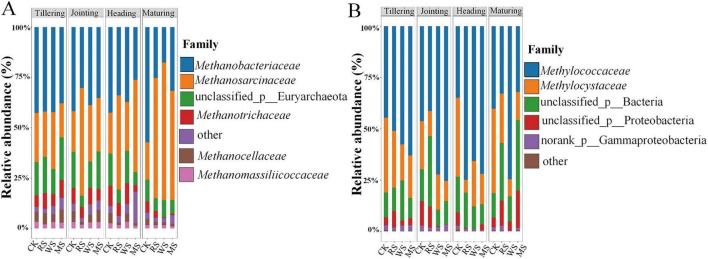
Community composition of methanogen **(A)** and methanotroph **(B)** under different treatments in various growth stages.

High-throughput sequencing of soil samples from successive rice growth stages identified 10 classes, 15 orders, 25 families, 38 genera, and 83 species of methanogenic archaea (Figure 3B). At the family level, *Methanobacteriaceae* (18–57%), *Methanosarcinaceae* (17–60%), unclassified_p__Euryarchaeota (6–21%), *Methanotrichaceae* (1–10%), *Methanocellaceae* (1–7%), and *Methanomassiliicoccaceae* (1–3%) dominated (Figure 3A). These families were categorized by metabolic pathway into hydrogenotrophic methanogens (*Methanobacteriaceae*, *Methanocellaceae*, *Methanomassiliicoccaceae*), obligate acetoclastic methanogens (*Methanotrichaceae*, *Methanosarcinaceae*).

Similarly, methanotrophic bacteria were classified into 6 classes, 11 orders, 12 families, 22 genera, and 43 species (Figure 3B). The predominant families included *Methylococcaceae* (32–75%), *Methylocystaceae* (6–41%), unclassified_d__*Bacteria* (8–34%), unclassified_p__Proteobacteria (1–18%), and norank_c__Gammaproteobacteria (1–3%). These taxa were grouped according to their carbon assimilation pathways into Type I (*Methylococcaceae*) and Type II (*Methylocystaceae*) methanotrophs.

**FIGURE 4:**
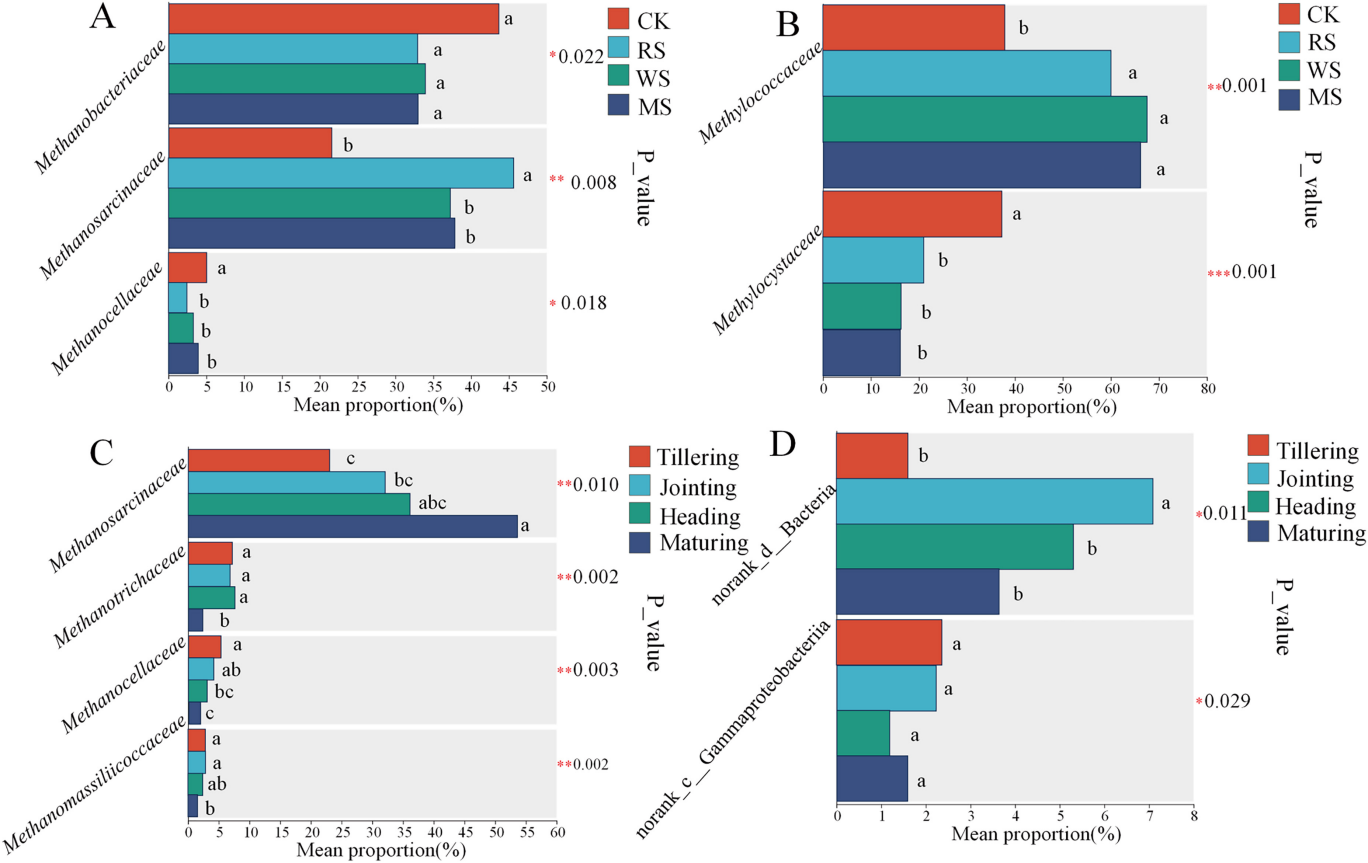
Comparative analysis of methanogenic **(A,B)** and methanotrophic **(C,D)** community composition at the family level under different treatments and growth stages. *, **, *** represent significant differences between treatments with *P* < 0.05, 0.01, and 0.001, respectively.

Straw incorporation (RS, WS, MS) significantly affected the relative abundance of dominant families ( ≥ 1%) in methanogenic and methanotrophic communities (*P* < 0.05) (Figure 4). Compared with CK, the overall pattern indicated increased representation of acetoclastic methanogens (*Methanosarcinaceae*) and decreased representation of key hydrogenotrophic lineages (*Methanocellaceae*) (Figure 4A). For methanotrophs, straw incorporation corresponded to higher Type I (*Methylococcaceae*) and lower Type II (*Methylocystaceae*) methanotrophs (Figure 4B).

Across growth stages, methanogenic communities exhibited treatment-dependent variation (Supplementary Figure 6). The most prominent differences involved shifts between acetoclastic-associated families and hydrogenotrophic-associated families. For example, at tillering, WS showed higher *Methanosarcinaceae* and lower *Methanocellaceae* than MS (*P* < 0.05), whereas stage-dependent reversals in *Methanocellaceae* were observed later (higher under WS at heading; *P* < 0.05). At maturity, RS showed higher *Methanotrichaceae* than WS and MS (*P* < 0.05), while WS and MS remained lower than CK (*P* < 0.001). Overall, *Methanosarcinaceae* increased from tillering to maturing, whereas *Methanocellaceae* showed the opposite trend (Figure 4C). In contrast, methanotroph composition varied less across stages, and no clear successional pattern was observed (Supplementary Figure 6).

### 3.5 LEfSe analysis of methanogens and methanotrophs

LEfSe analysis was employed to identify key biomarkers that significantly contributed to the compositional divergence in methanogenic and methanotrophic communities across treatments and growth stages. Using a “one-against-all” strategy, the *mcrA*-based methanogenic communities showed distinct biomarker separation among CK, RS, and MS treatments (LDA > 2), whereas no WS-specific methanogen biomarkers were detected under the applied LEfSe criteria.

**FIGURE 5:**
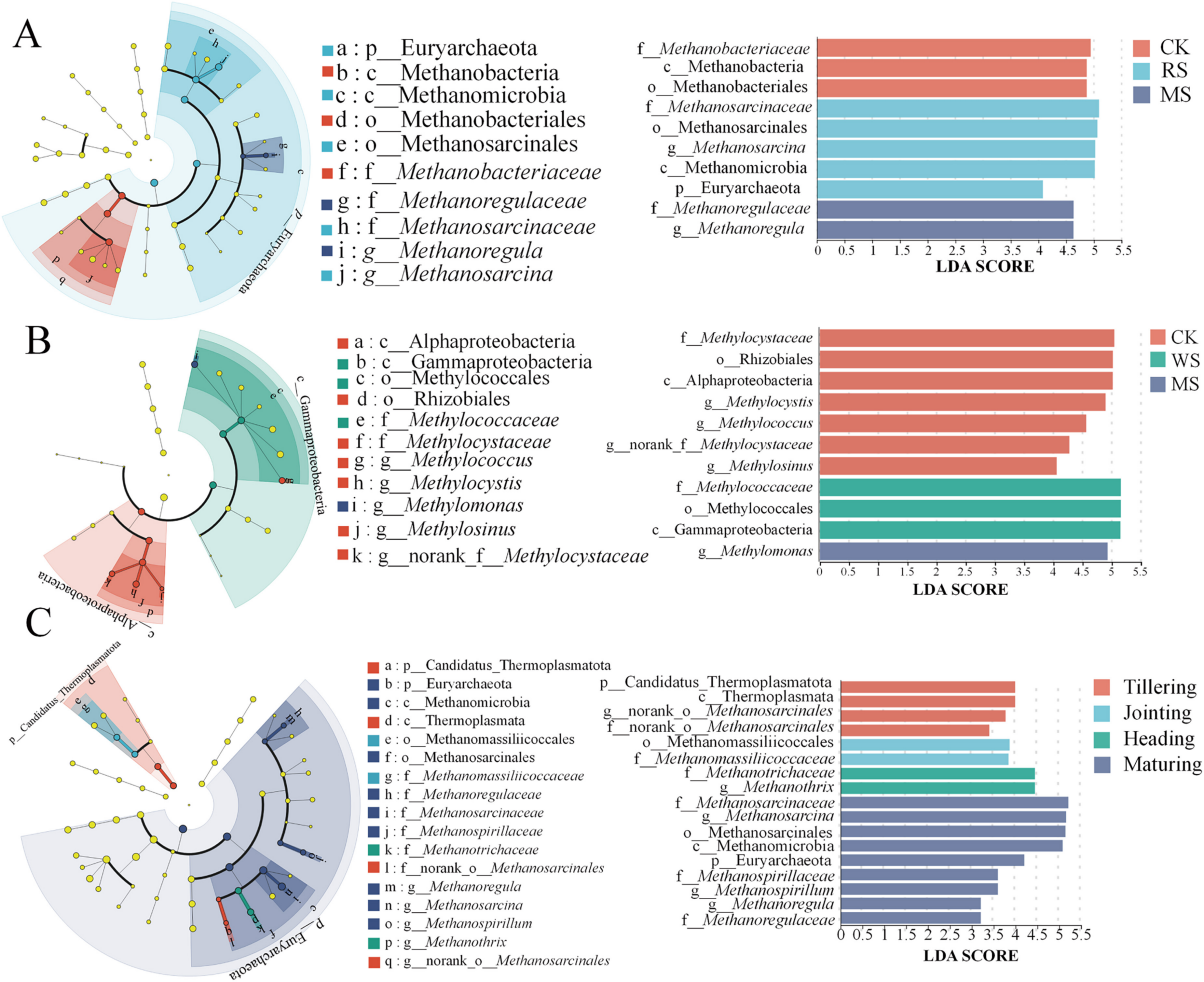
LEfSe analysis of differentially abundant biomarkers in methanogenic **(A,C)** and methanotrophic **(B)** communities under different straw treatments and rice growth stages.

Specifically, CK was characterized by Methanobacteria class, Methanobacteriales order and *Methanobacteriaceae* family as primary discriminators (LDA > 4.5; Figure 5A), signifying their diagnostic role in control ecosystems. In contrast, RS treatment featured Euryarchaeota phylum, Methanomicrobia class, Methanosarcinales order, *Methanosarcinaceae* family and *Methanosarcina* genus as key differentiators (LDA > 5), revealing acetoclastic pathway dominance as a signature response to rice straw. MS treatment prioritized *Methanoregulaceae* family and *Methanoregula* genus as defining biomarkers (LDA > 4.5). For methanotrophs (Figure 5B), WS treatment was discriminated by *Methylococcaceae* family, Methylococcales order, and Gammaproteobacteria class as high impact biomarkers (LDA > 5), consistent with a community compositional shift toward Type I methanotrophs. Meanwhile, MS treatment exhibited *Methylomonas* genus as its defining biomarker (LDA = 4.9).

Distinct successional discriminators emerged across rice growth stages (Figure 5C). During the tillering stage, four taxa served as key stage specific biomarkers including Candidatus*_*Thermoplasmatota phylum, Thermoplasmata class, alongside unclassified genus and family within the Methanosarcinales order. The jointing stage was characterized by Methanomassiliicoccales class and *Methanomassiliicoccaceae* family as phase specific differentiators. The heading stage featured *Methanotrichaceae* family and *Methanothrix* genus as primary temporal discriminators. By the maturity stage, nine taxonomic units as robust developmental biomarkers: Euryarchaeota phylum, Methanomicrobia class, Methanosarcinales order, *Methanosarcinaceae* family, *Methanospirillaceae* family, *Methanosarcina* genus, *Methanospirillum* genus, *Methanoregulaceae* family, *Methanoregula* genus.

### 3.6 RDA and correlation analyses of methanogens and methanotrophs with soil properties

**FIGURE 6:**
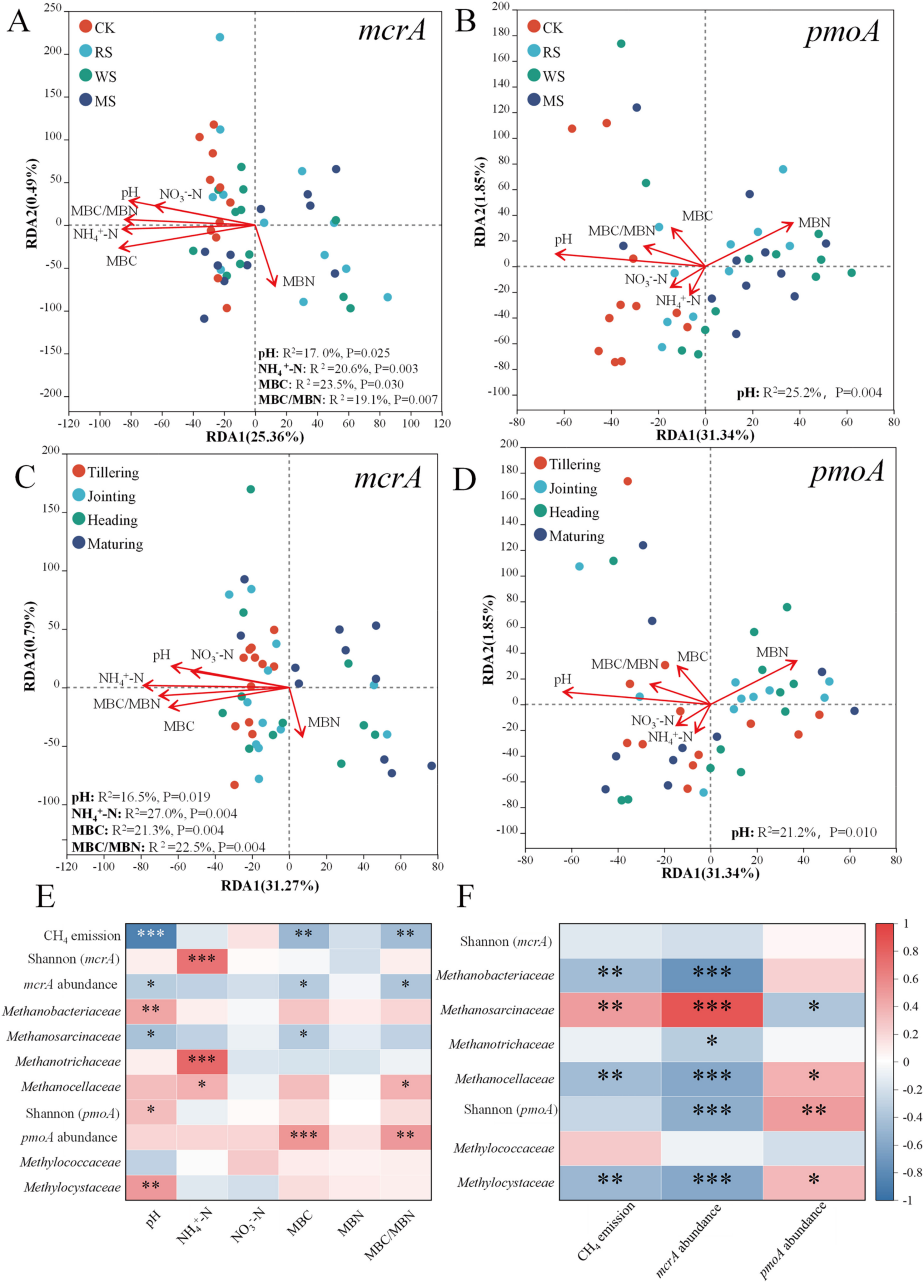
Redundancy analysis of the influence of soil physicochemical properties on methanogenic **(A,C)** and methanotrophic **(B,D)** community composition at the family level under different treatments **(A,B)** and growth stages **(C,D)**. Heatmap of correlations between environmental factors, CH_4_ emissions, and abundance, diversity, and community composition under different straw treatments **(E,F)**. *, **, and *** indicate significant differences at *P* < 0.05, *P* < 0.01, and *P* < 0.001, respectively.

Redundancy analysis (RDA) analysis showed that, irrespective of straw incorporation treatments or rice growth stages, the same suite of soil properties governed methanogenic community composition, although the exact *P*-values and proportions of explained variance differed slightly (Figure 6). In particular, MBC, NH_4_^+^–N, the MBC/MBN ratio and pH together accounted for the majority of variation in methanogenic assemblages of treatments and growth stages, with MBC explained 23.5% and 21.3% of the total variance (*P* = 0.03,*P* = 0.004), NH_4_^+^–N explaining 20.6% and 27.0% (*P* = 0.003,*P* = 0.004), the MBC/MBN ratio explaining 19.1% and 22.5% (*P* = 0.007,*P* = 0.004) and pH explaining 17.0% and 16.5% (*P* = 0.025,*P* = 0.019). Methanotrophic communities, by contrast, were consistently dominated by pH, which accounted for 25.2% and 21.2% of their variance (*P* = 0.004, *P* = 0.01) across all treatments and growth stages.

Pearson correlation analysis revealed that both CH_4_ emissions and *mcrA* gene copies were significantly negatively correlated with soil pH, MBC, and the MBC/MBN ratio (Figure 6E). Specifically, soil pH was strongly inversely correlated with CH_4_ emissions (*P* < 0.001), *mcrA* gene copies (*P* < 0.05), and *Methanosarcinaceae* abundance (*P* < 0.05), but positively correlated with *Methanobacteriaceae* (*P* < 0.01), the Shannon index of *pmoA* gene (*P* < 0.05), and *Methylocystaceae* (*P* < 0.01). MBC inversely correlated significantly with CH_4_ emissions (*P* < 0.01), *mcrA* copies (*P* < 0.05), and *Methanosarcinaceae* (*P* < 0.05), and was also positively correlated with *pmoA* gene copies (*P* < 0.001). The MBC/MBN ratio showed inverse correlations with CH_4_ emissions (*P* < 0.01) and *mcrA* gene copies (*P* < 0.05), yet was positively associated with *Methanocellaceae* (*P* < 0.05) and *pmoA* gene copies (*P* < 0.01). Notably, CH_4_ emissions themselves were positively correlated with *Methanosarcinaceae* (*P* < 0.01), and inversely correlated with *Methanobacteriaceae* (*P* < 0.05), *Methanocellaceae* (*P* < 0.05), and *Methylocystaceae* (*P* < 0.05) (Figure 6F).

### 3.7 PLS-PM analysis of factors influencing CH_4_ emissions

**FIGURE 7:**
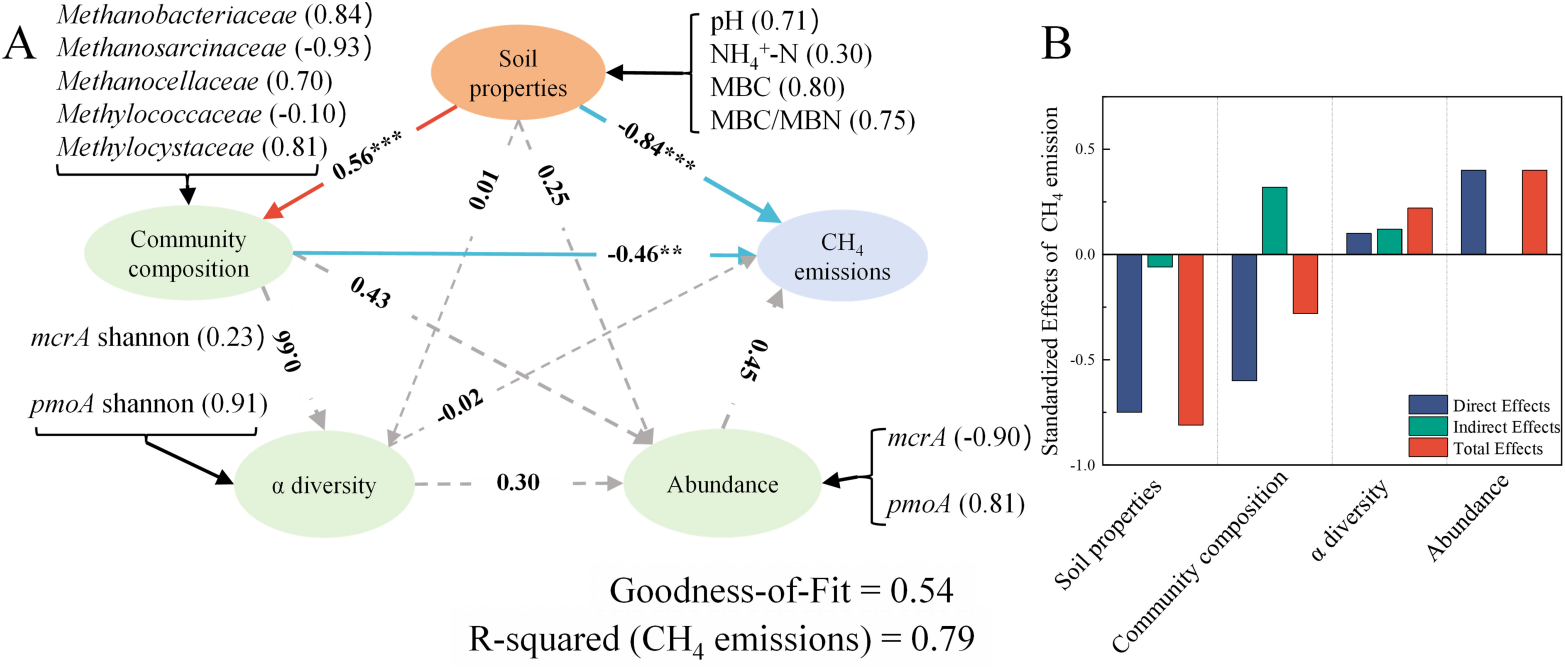
Partial least squares path modeling integrating CH_4_ emissions with soil properties, α-diversity, community composition (Key CH_4_-Cycling Taxa), and microbial abundance. Arrows indicate directional path relationships **(A)**. Blue and red arrows denote positive and negative effects, respectively, while solid and dashed arrows represent statistically significant and non-significant relationships (**P < 0.01, ***P < 0.001). The Goodness-of-Fit (GoF) index assesses the model’s overall fit to observed data, the robustness of individual paths was further verified by 95% Bootstrap confidence intervals. Standardized effects of soil properties, community composition, α-diversity, and microbial abundance on CH_4_ emission, showing direct, indirect, and total effects **(B)**.

Quantitative path analysis using PLS-PM revealed that CH_4_ emissions were jointly modulated by four latent variable domains (Figure 7A). Some pathways showed significant *P*-values in *T*-tests (*P* < 0.05) but included 0 in their Bootstrap confidence intervals. To ensure the reliability of conclusions, only pathways with Bootstrap confidence intervals excluding 0 were considered robust and included in the core interpretation. Explanatory power of latent variables is provided in the Supplementary Materials (Supplementary Table 5). Among these, soil properties (path coefficient = −0.84, *P* < 0.001) and microbial community composition (path coefficient = −0.46, *P* < 0.01) exerted significant negative effects on CH_4_ emissions. For the soil properties under different straw treatments, MBC (0.80) was the biggest factor for the changes, followed by the MBC/MBN (0.75) and pH (0.71). The microbial community mostly went up and down with the abundance of *Methanosarcinaceae* (−0.93), *Methanobacteriaceae* (0.84), *Methylocystaceae* (0.81), and *Methanocellaceae* (0.70).

## 4 Discussion

### 4.1 Effects of straw incorporation on CH_4_ emissions and key factors

Straw incorporation to cropland represents a sustainable management strategy that promotes the recycling of crop residues, improves soil fertility, and contributes to agricultural carbon sequestration and climate change mitigation (Zhao et al., 2024). Nevertheless, straw incorporation often leads to pronounced increases in CH_4_ emissions (Jiang et al., 2019; Ma et al., 2024; Qin et al., 2025). A similar trend was observed in the present study, where all three types of straw significantly increased cumulative CH_4_ emissions compared with CK (Figure 1A).

CH_4_ emissions were regulated by both abiotic and biotic factors. PLS-PM indicated that changes in soil properties caused by straw incorporation exerted the strongest influence on CH_4_ emissions, while microbial communities had a secondary effect; overall, the PLS-PM model accounted for 79% of the observed variation in CH_4_ emissions (Figure 7A). In addition, the direct effect of abiotic factors (soil properties) on CH_4_ emissions was stronger than that of biotic factors (Figure 7B). Previous studies have shown that straw incorporation increases CH_4_ emissions in paddy soils primarily by providing additional carbon substrates and altering soil physicochemical conditions that favor methanogenesis (Yuan et al., 2014; Jia et al., 2024). Recent structural equation modeling studies further suggest that soil carbon pool properties and associated soil biological activity mediate the effect of straw return on CH_4_ production pathways, highlighting the central role of changes in soil properties in driving methane emissions (Qin et al., 2025). Straw incorporation alters the abundance and composition of methanogens and methanotrophs, modulating methane production and oxidation, emphasizing that changes in soil properties and microbial responses drive CH_4_ emission variations (Bao et al., 2014; Ma et al., 2020; Yang et al., 2022).

### 4.2 Effects of straw incorporation on soil properties

Soil properties exerted a strong regulatory influence on CH_4_ emissions (Figure 7A); notably, soil pH, MBC, and the MBC/MBN ratio were all significantly negatively correlated with CH_4_ emissions (Figure 6E). Previous studies have reported that soil pH and MBC are important drivers of CH_4_ emissions in rice paddies (Quilliam et al., 2013; Shah et al., 2024; Lasar et al., 2025). Although straw C/N ratio is widely used as an indicator of residue quality because it helps predict whether decomposition is more likely to release mineral N (typically <20) or to temporarily immobilize soil N (often >30), this relationship is not always consistent across residues and conditions. Accordingly, our data do not support attributing among-straw differences in seasonal cumulative CH_4_ emissions to C/N alone (Trinsoutrot et al., 2000; Oliveira et al., 2020). In our experiment, the three straws had C/N ratios of 26.4–36.6 (Supplementary Table 1), while seasonal cumulative CH_4_ emissions did not differ significantly among RS, WS and MS. Under flooded conditions, straw incorporation commonly results in soil acidification, mainly through the accumulation of readily degradable organic acids produced during straw decomposition and the enhanced dissolution of CO_2_, thereby causing a decline in soil pH (Somboon et al., 2024). The soluble fractions of crop residues, structural carbon components (cellulose, hemicellulose, and lignin), and the C/N ratio collectively regulate decomposition dynamics and the temporal pattern of carbon release (Weiler et al., 2021; Sandhu et al., 2022). The greater pH decline under wheat straw incorporation, compared with rice straw, may reflect differences in residue quality and associated decomposition processes. Previous studies have shown that wheat straw decomposition can be accompanied by relatively greater accumulation of organic acid intermediates than rice straw, which is consistent with stronger soil pH decreases (Shan et al., 2008). By contrast, the comparatively weaker acidification observed under RS and MS may be explained by a partial buffering effect of soil mineral components, as suggested by studies showing that residue-derived acidity can be moderated by soil mineral reactions and base cation release during decomposition (Do et al., 2020; Puri et al., 2024).

The MBC/MBN has been proposed as a management-sensitive indicator of soil quality processes and as a useful metric for identifying management practices associated with changes in rice paddy productivity (Li et al., 2016). Straw incorporation can alter soil organic matter mineralization dynamics by rapidly consuming readily available substrates early on and slowing as substrates become depleted (Liu et al., 2024). Because substrate and nutrient availability regulate microbial growth, microbes may allocate more carbon to biomass rather than respiration under nutrient-sufficient conditions, which may lead to asynchronous changes in MBC and MBN (Singh et al., 2024). Notably, mean MBC did not differ significantly among straw treatments, suggesting that its influence is mainly stage-dependent rather than treatment-driven. In line with our results, MBC showed the highest loading within the soil-properties domain in the PLS-PM (Figure 7A) and was negatively correlated with CH_4_ emissions (Figure 6E), suggesting that microbial biomass dynamics may modulate the fraction of straw-derived carbon ultimately channeled into methanogenesis (Schimel and Schaeffer, 2012; Wang et al., 2022).

### 4.3 Effects of straw incorporation on methanogenic communities

No WS-specific methanogen biomarkers were detected under the applied LEfSe criteria. This absence does not necessarily indicate that methanogens were absent in WS, but rather that none showed a consistent and sufficiently large enrichment that met LEfSe’s combined requirements of statistical significance, biological consistency, and effect relevance (LDA effect size) (Segata et al., 2011). A field study reported that under straw incorporation into non-native soil, the straw C decomposition rate followed corn > wheat > rice over 270 days, demonstrating different decomposition dynamics among crop residues (Wang et al., 2017). However, under anoxic paddy-soil conditions, straw amendment can rapidly generate a broadly similar set of fermentation intermediates (e.g., acetate, propionate, and butyrate) that directly fuel methanogenesis, and downstream methanogenic pathways may converge even when different straw fractions are supplied (Glissmann and Conrad, 2000). Such shared intermediary metabolism can lead to overlapping community responses across straw treatments, reducing the likelihood that any WS-specific taxon emerges as a robust LEfSe biomarker.

In this study, soil properties directly influenced CH_4_ emissions and indirectly shaped the methanogenic community (Figure 7A). Accordingly, CH_4_ emissions were significantly positively correlated with the dominant family *Methanosarcinaceae* (Figure 6F). The relative abundance of *Methanosarcinaceae* in the WS treatment was significantly lower than that in RS, but still higher than in CK (Figure 4A). Meanwhile, *Methanosarcinaceae* was significantly negatively correlated with soil pH and MBC (Figure 6E), indicating that straw-induced decreases in soil pH and associated shifts in microbial biomass co-occurred with the enrichment of this family. Importantly, *Methanobacteriaceae* was lower in the straw incorporation treatments than in CK (Figure 4A), and CH_4_ emissions were significantly negatively correlated with *Methanobacteriaceae* (Figure 6F). Collectively, these results suggest that straw incorporation shifted the dominant methanogenic community from hydrogenotrophic *Methanobacteriaceae* toward *Methanosarcinaceae*. Notably, although *Methanosarcinaceae* is often categorized as an acetoclastic methanogenic lineage (Conrad et al., 2012), it is metabolically versatile; compared with hydrogenotrophic *Methanobacteriaceae*, *Methanosarcinaceae*—especially the genus *Methanosarcina*—can utilize multiple methanogenic pathways, including acetoclastic, methylotrophic, and, in some cases, hydrogenotrophic methanogenesis (Wintsche et al., 2018). Straw incorporation introduces exogenous organic matter, which can be converted through fermentative bacterial hydrolysis and acidogenesis into H_2_, acetate, and other fermentation products (Glissmann and Conrad, 2000; Baba et al., 2016). The increased availability of these substrates may favor the enrichment of *Methanosarcinaceae*, which can exploit diverse precursors to produce CH_4_ (Lu et al., 2015; Alpana et al., 2017; Zhou et al., 2020a).

### 4.4 Effects of straw incorporation on methanotrophic communities

On the oxidation side, CH_4_ emissions were significantly negatively correlated with the dominant family *Methylocystaceae* (Figure 6F), and straw incorporation significantly reduced *Methylocystaceae* compared with CK (Figure 4B). In addition, *Methylocystaceae* was significantly positively correlated with soil pH (Figure 6E). This is consistent with evidence that soil pH is a key environmental filter structuring canonical Type I versus Type II methanotroph assemblages in paddy soils (Zhao et al., 2020). In contrast, straw incorporation significantly increased *Methylococcaceae* relative to CK (Figure 4B), indicating that the dominant methanotrophic community shifted from Type II methanotrophs (*Methylocystaceae*) toward Type I methanotrophs (*Methylococcaceae*) in straw-amended paddy soils. In flooded paddy soils, aerobic CH_4_ oxidation is typically confined to a thin oxic–anoxic interface, and even millimeter-scale oxygen gradients can strongly affect methanotroph distribution and activity (Reim et al., 2012). Previous work has also suggested that as CH_4_ concentrations increase, Type I methanotrophs may become dominant, although their oxidation efficiency is not necessarily higher than that of Type II methanotrophs (Shiau et al., 2018). Meanwhile, the overall differences in *pmoA* copy numbers among treatments were relatively small (Figure 1E), implying that CH_4_-oxidation capacity did not increase in parallel (Tentori Egidio and Richardson Ruth, 2020). Thus, the observed community shift may have weakened the oxidative buffer, insufficiently offsetting the stimulated methanogenesis, leading to higher net emissions.

### 4.5 Implications and limitations

From a management perspective, WS showed the largest yield increase, while its seasonal CH_4_ increase was lower than MS and comparable to RS (Figure 1A). Although the yield differences among straw types were not statistically significant, this pattern suggests that wheat straw incorporation may offer a favorable productivity–emissions trade-off under the tested conditions. Further multi-season field trials are needed to confirm whether this advantage persists across soil types and climatic regimes. A key limitation is that we did not measure DOC or fermentation intermediates (acetate and other short-chain fatty acids), which would directly connect straw decomposition to methanogenic substrate supply. We also did not quantify redox potential or micro-scale O_2_ gradients that constrain CH_4_ oxidation at the oxic–anoxic interface. Future work integrating these measurements with potential-rate assays and residue biochemical characterization would provide stronger mechanistic attribution of straw-type effects on net CH_4_ emissions.

## 5 Conclusion

Straw incorporation (rice, wheat, and maize) significantly increased seasonal CH_4_ emissions compared with the no-straw control under the same fertilization and water regime. However, seasonal cumulative CH_4_ emissions did not differ significantly among the three straw types. Across treatments, soil pH was the only soil property showing a consistent treatment effect and was closely associated with the structure of CH_4_-cycling communities. Straw incorporation were accompanied by a methanogenic shift toward *Methanosarcinaceae* and a methanotrophic shift from Type II (*Methylocystaceae*) toward Type I (*Methylococcaceae*), while *pmoA* gene abundance showed weak treatment differentiation, suggesting limited oxidative compensation at the community level. Wheat straw showed a numerically higher yield increase, while seasonal CH_4_ emissions did not differ significantly among straw types. These results suggest a potential productivity–emissions advantage of wheat straw incorporation, which warrants further verification. Future work should quantify dissolved organic carbon, key fermentation intermediates, and redox and oxygen dynamics, and explicitly test pH amendments and fertilizer management for their effects on CH_4_ emissions in field trials.

## Data Availability

The datasets presented in this study can be found in online repositories. The names of the repository/repositories and accession number(s) can be found below: https://www.ncbi.nlm.nih.gov/, PRJNA1254631, https://www.ncbi.nlm.nih.gov/, PRJNA1254505.

## Footnotes

1
http://drive5.com/uparse/

2
http://rdp.cme.msu.edu/

3
https://cloud.majorbio.com
