# Supplementary material for: Straw type governs methane-cycling microbiomes and CH4 emissions in paddy soils via abiotic and biotic interactions
Source: Front Microbiol. 2026 Jan 16;16:1750602. doi: 10.3389/fmicb.2025.1750602 (PMC12855542; doi:10.3389/fmicb.2025.1750602)
Supplement: Supplementary file 1 [file Supplementary_file_1.docx]

**Supplementary materials methods**

1. DNA extraction and PCR amplification

At four key growth stages of rice (tillering, jointing, heading, and maturing), 0.5 g of surface soil samples were collected. Genomic DNA was extracted using the Fast DNA SPIN Kit (MP Biomedicals, Eschwege, Germany). The quality and concentration of DNA were determined by 1.0% agarose gel electrophoresis and a NanoDrop2000 spectrophotometer (Thermo Scientific, United States) and kept at -80℃ prior to further use.

Using the extracted DNA as a template, the *pmoA* gene was amplified by PCR with primers A189f/Mb661R tagged with barcode sequences, and the *mcrA* gene was amplified with primers MLfF/MLrR carrying barcode sequences. PCR amplification cycling conditions were as follows: initial denaturation at 95 ℃ for 3 min, followed by 27 cycles of denaturing at 95℃ for 30 s, annealing at 55 ℃ for 30 s and extension at 72 ℃for 45 s, and single extension at 72 ℃ for 10 min, and end at 4 ℃. Amplicons were separated on 2% agarose gels, excised, and purified using a PCR Clean-Up Kit (YuHua, Shanghai, China). Purified products were quantified with a Qubit 4.0 fluorometer (Thermo Fisher Scientific, USA).

1. Illumina sequencing and data processing

Purified PCR products were used to construct sequencing libraries with the NEXTFLEX Rapid DNA-Seq Kit (PerkinElmer). Library preparation comprised four steps: (1) adapter ligation; (2) removal of adapter dimers by magnetic-bead cleanup; (3) PCR amplification to enrich adapter-ligated fragments; (4) final purification with magnetic beads to yield the completed library. Libraries were sequenced on an Illumina NextSeq 2000 platform (Shanghai Meiji Biomedical Technology Co., Ltd.). The sequencing libraries achieved coverage of more than 99%, confirming that the sequencing depth was sufficient to capture the microbial community structure and that the results are highly representative. The raw data have been deposited in the NCBI Sequence Read Archive under the accession numbers PRJNA1254631 (*mcrA* gene) and PRJNA1254505 (*pmoA* gene).

Paired-end raw sequencing reads were quality-controlled using fastp (version 0.19.6; <https://github.com/OpenGene/fastp>) ([He et al., 2020](#_ENREF_14)), and merged using FLASH (version 1.2.11; <http://www.cbcb.umd.edu/software/flash>). The processing steps were as follows: (1) bases with a quality score below 20 at the read ends were filtered using a 50 bp sliding window; if the average quality score within the window dropped below 20, bases from that point onward were trimmed; reads shorter than 50 bp after trimming and those containing ambiguous “N” bases were removed; (2) paired-end reads were merged into single sequences based on their overlapping regions, with a minimum overlap length of 10 bp; (3) a maximum mismatch ratio of 0.2 was allowed in the overlap region, and sequences not meeting this criterion were excluded; (4) sequences were assigned to individual samples based on exact barcode matches (0 mismatches allowed) and primer recognition (up to 2 mismatches allowed), and sequence orientation was adjusted accordingly.

High-quality merged sequences were clustered into operational taxonomic units (OTUs) at 97% sequence similarity using UPARSE v7.1 (<http://drive5.com/uparse/>), with chimera sequences removed during clustering. To minimize the influence of sequencing depth on subsequent alpha and beta diversity analyses, all samples were rarefied to the same sequencing depth based on the smallest library size. After rarefaction, the average Good’s coverage per sample remained at 99.98%. Taxonomic classification of *pmoA* and *mcrA* gene OTUs was conducted using the RDP Classifier (version 2.11; <http://rdp.cme.msu.edu/>) against the fgr/*pmoA*_202012 and fgr/*mcrA*_202012 reference databases, with a confidence threshold of 70%. Community composition was then summarized across different taxonomic levels for each sample.

**Supplementary Tables and Figures**

**Table S1** Chemical properties of tested straw

| Type | TN% | TP% | TK% | TC% | C/N |
| --- | --- | --- | --- | --- | --- |
| RS | 1.49±0.08a | 0.21±0.02b | 1.54±0.12b | 39.30±0.05a | 26.35±1.12b |
| WS | 1.22±0.08b | 0.07±0.01c | 0.91±0.01c | 36.70±0.14b | 30.06±2.10ab |
| MS | 1.04±0.07c | 1.98±0.00a | 1.98±0.00a | 36.00±0.14b | 34.59±2.37a |

Data are presented as the mean ± standard deviation. Different lowercase letters in the same column indicate a significant difference among different treatments.

**Table S2** Summary of normality and homogeneity of variance tests for key response variables

| **Variable** | **Growth Stage** | **Normality** | **Homogeneity of Variance** |
| --- | --- | --- | --- |
| pH | Tillering | Yes | No |
|  | Jointing | Yes | Yes |
|  | Heading | Yes | Yes |
|  | Maturing | No | No |
| MBC | Tillering | Yes | Yes |
|  | Jointing | Yes | Yes |
|  | Heading | No | Yes |
|  | Maturing | Yes | Yes |
| MBN | Tillering | No | Yes |
|  | Jointing | No | Yes |
|  | Heading | No | Yes |
|  | Maturing | Yes | Yes |
| MBC/MBN | Tillering | Yes | Yes |
|  | Jointing | Yes | No |
|  | Heading | No | Yes |
|  | Maturing | Yes | No |
| *mcrA* | Tillering | No | Yes |
|  | Jointing | Yes | Yes |
|  | Heading | Yes | No |
|  | Maturing | Yes | Yes |
| *pmoA* | Tillering | Yes | No |
|  | Jointing | Yes | Yes |
|  | Heading | No | Yes |
|  | Maturing | Yes | No |

**Table S3** α-diversity indices of methanogenic community

| Growth Stage | Treatment | Richness index | | Diversity index | | shannoneven |
| --- | --- | --- | --- | --- | --- | --- |
|  |  | chao1 | ace | shannon | simpson |  |
| Tillering | CK | 15±1aA | 15.2±1.31aA | 1.66±0.03bA | 0.26±0.01abA | 0.62±0.02aA |
|  | RS | 15.67±1.53aA | 16.08±1.82aA | 1.68±0.05abA | 0.25±0.03abA | 0.61±0.03aA |
|  | WS | 16±1aA | 16.13±1.2aA | 1.64±0.1bA | 0.28±0.02aB | 0.59±0.04aA |
|  | MS | 16.33±1.53aA | 16.62±2aA | 1.86±0.09aA | 0.21±0.02bA | 0.67±0.02aA |
| Jointing | CK | 16±1aA | 16.62±1.08aA | 1.75±0.17aA | 0.25±0.06bA | 0.63±0.07aA |
|  | RS | 17.83±1.26aA | 18.29±1.01aA | 1.21±0.25bA | 0.44±0.11aA | 0.42±0.08bA |
|  | WS | 18±3.46aA | 18.93±5.08aA | 1.72±0.12aA | 0.25±0.03bB | 0.6±0.06aA |
|  | MS | 16.33±0.58aA | 16.33±0.58aA | 1.81±0.06aA | 0.22±0.01bA | 0.65±0.02aA |
| Heading | CK | 15.67±1.53aA | 15.94±1.75aA | 1.73±0.1aA | 0.25±0.04aA | 0.63±0.05aA |
|  | RS | 18.67±4.04aA | 18.56±3.6aA | 1.28±0.18aA | 0.4±0.05aA | 0.45±0.09aA |
|  | WS | 17.33±1.53aA | 17.55±1.88aA | 1.78±0.07aA | 0.24±0.01aB | 0.63±0.03aA |
|  | MS | 18±2.65aA | 18.39±2.39aA | 1.43±0.43aA | 0.36±0.14aA | 0.49±0.13aA |
| Maturing | CK | 18.67±2.31aA | 18.83±2.46aA | 1.41±0.3aA | 0.39±0.14aA | 0.49±0.12aA |
|  | RS | 17.08±2.79aA | 17.56±3.61aA | 1.14±0.44aA | 0.48±0.22aA | 0.4±0.15aA |
|  | WS | 15±0aA | 15.12±0.2aA | 1.08±0.07aB | 0.51±0.01aA | 0.4±0.03aB |
|  | MS | 16.4±0.53aA | 17.4±0.64aA | 1.21±0.4aA | 0.42±0.13aA | 0.43±0.15aA |
| *P* | Treatment | 0.667 | 0.760 | **0.0001***** | **0.0001***** | **0.0001***** |
|  | Growth Stage | 0.219 | 0.299 | **0.0099**** | **0.035*** | **0.006**** |
|  | Treatment  ×Growth Stage | 0.339 | 0.479 | 0.099 | 0.202 | 0.098 |

**Table S4** α-diversity indices of methanotrophic community

| Growth Stage | Treatment | Richness index | | Diversity index | | shannoneven |
| --- | --- | --- | --- | --- | --- | --- |
|  |  | chao | ace | shannon | simpson |  |
| Tillering | CK | 9±0aA | 9±0aA | 1.28±0.12aA | 0.35±0.05aA | 0.58±0.05aA |
|  | RS | 9.33±0.58aA | 9.53±0.92aA | 1.25±0.1aA | 0.36±0.05aB | 0.56±0.04aA |
|  | WS | 8±1.73aA | 9±0aA | 0.94±0.37aA | 0.52±0.23aA | 0.47±0.22aA |
|  | MS | 9.33±0.58aA | 10.38±0.54aA | 1.02±0.4aA | 0.5±0.21aA | 0.46±0.18aA |
| Jointing | CK | 9±0aA | 10±0.94aA | 1.22±0.41aA | 0.4±0.23aA | 0.56±0.19aA |
|  | RS | 7.67±2.31aA | 8.29±1.23aA | 0.93±0.25aA | 0.49±0.07aAB | 0.46±0.06aA |
|  | WS | 8±1aA | 8.62±2.01aA | 0.83±0.3aA | 0.59±0.19aA | 0.4±0.13aA |
|  | MS | 8.67±0.58aA | 8.85±0.27aA | 0.85±0.32aA | 0.6±0.17aA | 0.39±0.14aA |
| Heading | CK | 9±1aA | 9±0aA | 1.32±0.14aA | 0.33±0.06bA | 0.6±0.09aA |
|  | RS | 7±2.65aA | 7.84±3.32aA | 0.78±0.22bA | 0.6±0.08aA | 0.41±0.03aA |
|  | WS | 8.67±0.58aA | 9.28±1.45aA | 0.96±0.11abA | 0.5±0.08abA | 0.44±0.04aA |
|  | MS | 7±2.65aA | 9.07±0.75aA | 0.85±0.09bA | 0.56±0.06aA | 0.48±0.18aA |
| Maturing | CK | 8.67±0.58aA | 8.67±0.58aA | 1.26±0.05aA | 0.36±0.03aA | 0.58±0.01aA |
|  | RS | 7.67±2.31aA | 7±2.83aA | 1.02±0.31aA | 0.44±0.1aAB | 0.51±0.09aA |
|  | WS | 5.67±2.52aA | 7±1.41aA | 0.71±0.71aA | 0.66±0.36aA | 0.39±0.39aA |
|  | MS | 8±1aA | 8±1.41aA | 1.11±0.4aA | 0.41±0.14aA | 0.53±0.18aA |
| *P* | Treatment | 0.207 | 0.487 | **0.021*** | **0.022*** | 0.117 |
|  | Growth Stage | 0.164 | 0.157 | 0.598 | 0.597 | 0.732 |
|  | Treatment  ×Growth Stage | 0.503 | 0.936 | 0.871 | 0.721 | 0.985 |

**Note:** Data are presented as the mean ± standard deviation. Different lowercase letters in the same column indicate significant differences between treatments within the same period, while uppercase letters denote significant differences across periods within the same treatment.

**Table S5** Latent Variable Explanatory Power

| Coefficient of Determination | Value | Description |
| --- | --- | --- |
| Soil Properties | 0 | Exogenous latent variable, no explanatory power assessment required |
| Community Composition | 0.31 | Moderate explanatory power |
| α-diversity | 0.44 | Moderate explanatory power |
| Abundance | 0.68 | Strong explanatory power |
| CH_4_ Emissions | 0.79 | Strong explanatory power |

**Figure. S1**

Seasonal variation in CH_4_ emission flux during rice growth season. Note: BF: Basal Fertilizer, TF: Tillering Fertilizer, PIF: Panicle Initiation Fertilizer, MSA: Mid-Season Aeration.

**Figure. S2**

Soil physicochemical properties. (A: ammonium nitrogen (NH_4_^+^–N); B: nitrate nitrogen (NO_3_^-^–N)).

**Figure. S3**

Average soil properties under different straw treatments. (A: pH; B: MBC; C: MBN; D: MBC/MBN; E: NH_4_^+^–N; F: NO_3_^-^–N).

**Figure. S4**

Rice grain yield under straw incorporation treatments. Different letters indicate significant differences among treatments (*P* < 0.05).

**Figure. S5**

Venn diagram showing unique and shared OTUs of methanogenic (A, C) and methanotrophic (B, D) communities under different treatments (A, B) and across growth stages (C, D).

**Figure. S6**

Differential analysis of the methanogenic archaeal community at the family level among different treatment groups across growth stages (A, B: tillering stage; C: jointing stage; D: heading stage; E, F, G: maturing stage).
